# Pruritus in hemodialysis patients

**DOI:** 10.1186/1471-5945-5-7

**Published:** 2005-06-24

**Authors:** Maryam Akhyani, Mohammad-Reza Ganji, Nasrin Samadi, Behnaz Khamesan, Maryam Daneshpazhooh

**Affiliations:** 1 Department of dermatology, Tehran University of Medical Sciences, Razi Hospital, Tehran, Iran; 2 Department of nephrology, Tehran University of Medical Sciences, Dr. Shariati hospital, Tehran, Iran; 3 Department of pathology, Tehran University of Medical Sciences, Sina hospital, Tehran, Iran; 4 Department of pediatrics, Iran University of Medical Sciences, Hazrat Rasool Hospital, Tehran, Iran

## Abstract

**Background:**

Pruritus is one of the most bothersome symptoms in patients on maintenance hemodialysis (HD), however little progress is seen in our understanding of its pathogenesis. The aim of this study was to evaluate the frequency of pruritus in HD patients in Tehran, Iran, and to correlate its presence and intensity with relevant clinical and laboratory parameters.

**Methods:**

One hundred sixty-seven patients on maintenance HD at three out-patient HD units were enrolled in the study. Itch intensity was scored as mild, moderate and severe. Some relevant clinical and laboratory parameters (age, sex, xerosis, presence of neuropathy, duration of dialysis, history of atopy and laboratory findings including hematocrit, creatinine, urea, calcium, phosphorus, parathyroid hormone [PTH] and alkaline phosphatase) were evaluated.

**Results:**

Pruritus was found in 41.9% of patients. The intensity of itching was mild, moderate and severe, in 51.4%, 11.4% and 37.7% of patients, respectively. In 22 patients (31.4%) pruritus intensified during and after dialysis. There was no significant difference in the serum levels of creatinine, blood urea nitrogen, calcium, phosphorus, alkaline phosphatase, PTH and hematocrit between patients with and without pruritus. Age, sex, xerosis, underlying renal disease, history of atopy and duration of haemodialysis were not significantly different between the two groups. However, neuropathy was significantly more common in the pruritic group (63.8% versus 42.1%) (pv = 0.006).

**Conclusion:**

Clinical neuropathy was the only significant finding in the pruritic group in our study. This finding justifies further research on nerve function and neurotransmitters in hemodialysis patients and the introduction of new drugs targeting neuropathy.

## Background

Pruritus often constitutes a major problem for patients with end-stage renal disease (ESRD). Unfortunately, dialysis has only a slight impact on pruritus. Therefore it is quite frustrating that an ever-increasing number of ESRD patients on HD, are waiting for transplantation, while they suffer from this tormenting symptom. According to most sources, more than half of patients undergoing HD complain of varying degrees of pruritus. [1-4] The mechanism underlying pruritus is poorly understood; current theories include secondary hyperparathyroidism, divalent-ion abnormalities, histamine, allergic sensitization, proliferation of skin mast cells, iron deficiency anemia, hypervitaminosis A, xerosis, neuropathy and neurologic changes, opioid system involvement (understimulation of κ receptors or overexpression of μ receptors), cytokines, serum bile acids, nitric oxide, or some combination of these.[1,4-12] The quality of uremic pruritus varies between patients. In some it is persistent, extensive and intractable but in others it may be transitory and localized. [1] Pruritus usually begins about six months after the start of dialysis, [13] and some authors have shown a significant positive relationship with the duration of HD. [10] The aim of this study was to evaluate the frequency of pruritus in hemodialysis patients in Tehran, Iran, and its relationship with age, sex, xerosis, neuropathy, duration of dialysis, history of atopy and laboratory findings including hematocrit, creatinine, urea, calcium, phosphorus, alkaline phosphatase and PTH.

## Methods

All patients (n = 167)on maintenance HD attending three HD units at Emam Khomeini, Dr Shariati and Sina Medical Complexes in Tehran, Iran, from 1998–1999 were enrolled in a cross-sectional study. Eighty-three were men and 84 were women. Their mean age was 47.95 +/-16.73 years with a range of 10–76 years. The routine of dialysis was 3 times per week (except holidays) for 4 hours. The mean of dialysis sessions per week are presented in table 1. Polysulfone membranes were used as dialyser, the dialysate was mainly acetate, and sodium hypochlorite was used for sterilization. Twenty nine of the patients were diabetic, while four patients suffered from systemic lupus erythematosus (SLE), two from lymphoma and one from multiple myeloma. The etiology in the remaining cases was unknown. None of the patients presented with primary skin disease.

**Table 1:** Comparison of clinical and laboratory parameters in HD patients

Clinical & laboratory parameters	Pruritic (mean ± SD)	Non pruritic (mean ± SD)	p-value
Age (years)	51.54 ± 16.63	45.37 ± 16.41	0.1212
Time on treatment (months)	44.42 ± 44	41.41 ± 44.73	0.666
Dialysis per week	2.6 ± 0.55	2.57 ± 0.59	0.682
Hematocrit (%) (M = 39–52)(F = 36–46)	25.43 ± 6.99	24.95 ± 5.43	0.647
Serum calcium (mg/dl) (8.6–10.3)	8.705 ± 1.79	8.707 ± 1.44	0.994
Serum phosphorus (mg/dl) (2.7–4.5)	5.45 ± 1.72	5.74 ± 1.66	0.314
Serum alkaline phosphatase (u/l) (150–450 u/l)	322.01 ± 272.61	494.56 ± 750.76	0.080
BUN (mg/dl) (8–20)	131.31 ± 66.01	136.08 ± 68.61	0.673
Serum creatinine (mg/dl) (0.4–1.3)	9.43 ± 3.21	9.27 ± 3.29	0.777
Parathyroid hormone (pmole/l)(0.8–2.5)	0.9 ± 1.16	1.43 ± 0.85	0.252

The patients were questioned, by a physician, if they had pruritus during the previous two weeks, and the symptoms were classified according to intensity (mild, moderate, and severe), location (generalized, head and neck, trunk and limbs), type of pruritus (episodic, persistence) and duration of HD. The patients' symptoms were recorded before a mid-week HD session and a blood sample was collected. The following laboratory tests were performed: Serum calcium, phosphorus, alkaline phosphatase, PTH, BUN, creatinine and hematocrit.

### Measurement of pruritus

The severity of pruritus was assessed subjectively and scored as follows:

Mild: Episodic and localized pruritus without disturbance in usual work and sleep.

Moderate: Generalized and continuous pruritus without sleep disturbance.

Severe: Generalized and continuous pruritus with sleep disturbance.

The major emphasis was on the disturbance in usual work and sleep.

### Grading of xerosis

Xerosis was graded as follows:

0: Absent; 1: Mild (only on legs); 2: Moderate (all of the extremities); and 3: Severe (generalized).

### Neuropathy

The diagnosis of clinical neuropathy was based on the presence of paresthesia, restless leg syndrome, decreased sensations of vibration, position, light touch, pain, decreased deep tendon reflexes or muscular force.

### Definition of atopy

Positive history of asthma, atopic dermatitis (based on Hanifin-Rajka criteria [14]) or allergic rhinitis was defined as atopy.

### Statistical analysis

Pruritus and non-pruritus groups were compared according to the variables. Data entry and coding was done through a database system developed with PE2 and analyzed by SPSS version 9. Student t-test and X^2 ^score were used for statistical analysis. Statistical significance was defined as p < 0.05.

## Results

Seventy patients (41.9%) (30 women and 40 men) complained of pruritus classified as mild, moderate and severe in 51.4% (n = 36), 11.4% (n = 8) and 37.1% (n = 26) of patients, respectively. The difference between the frequency of pruritus by gender was not significant (pv > 0.05). Pruritus was episodic in 60% (n = 42) and persistent in 40%(n = 28) of patients. Six patients had episodic but generalized pruritus with disturbance in work or sleep and their itch was categorized as moderate or severe. In 31.4% (n = 22) of patients pruritus intensified during and after dialysis.

Pruritus was generalized in 70 %(n = 49) of patients, limited to the trunk in 14.3% (n = 10), limbs 14.3% (n = 10), and head and neck 1.4 %(n = 1). There was history of atopy in 17 (24.3%) of pruritic and 20 (20.6%) of non-pruritic patients. The difference was not significant (pv > 0.05). Nine out of 21 diabetic patients, one out of 4 SLE patients and one lymphomatous patient complained of pruritus. There was no statistically significant difference between pruritus in diabetic and non-diabetic subjects (pv > 0.05). Forty seven out of 97 non-pruritic patients had a past history of pruritus during HD treatment. Comparison of clinical and laboratory parameters in HD patients with and without pruritus is shown in table 1. There was no significant difference among the two groups using t-test (pv > 0.05).

There wasn't any relationship between pruritus and xerosis in HD patients 2. Pruritus was alleviated in one patient after parathyroidectomy.

**Table 2:** Frequency of pruritus in HD patients according to severity of xerosis

Xerosis	Pruritic	Non pruritic
Absent	27 (38.6%)	33 (34%)
Mild	19 (27.1%)	40 (41.2%)
Moderate	19 (27.1%)	18 (18.6%)
Severe	5 (07.1%)	6 (06.2%)
Total	70 (100%)	97 (100%)

X^2 ^pv = 0.26

Eighty-four patients (51.3%) suffered from peripheral neuropathy (Three patients with diabetic foot amputation were excluded). Fifty-eight had only subjective symptoms; decreased sensation of vibration, decreased deep tendon reflexes, decreased pain perception, decreased sensation of light touch, and decreased sensation of position were noted in 19, 11, 7, 5, and 4 cases, respectively. In the pruritic group, 44 patients (63.8%), and in the non-pruritic group, 40 cases (42.1%) had neuropathy. The difference was significant (p-value = 0.006).

## Discussion

Pruritus is one of the most prevalent presentations of uremia and occurs in 10–85% of HD patients. [1,15-17] Difficulty in defining this very subjective symptom, the limited number of patients in most series, and the retrospective nature of some of the information, may be some of the reasons why such a wide range of figures is quoted. Recently, Urbonas et al noticed a decreasing trend in the prevalence of pruritus in HD patients and attributed it to more precise calculation of HD doses based on Kt/v or creatinine clearance measurements, introduction of new dialysers with larger surfaces as well as replacement of cuprophane fibers to more biocompatible ones made from polysulfone and amyl nitrite. [1,10,18]

Despite the long history of HD in Iran, to the best of our knowledge no study is published in the literature regarding the itch in Iranian HD patients. In our study, including 167 patients, pruritus was found in 41.9% of patients, severe in 37.1%, moderate in 11.4%, and mild in 51.4% of pruritic patients. Stahle-Backdahl et al, described pruritus in 60–65% of their cases which was severe in 8%, moderate in 24%, and mild in 34% of the examined patients. [19] Zucker and his colleagues reported pruritus in 48% of their HD patients at the time of the study in Tel-Aviv. [20] The figure reported from Poland by Szepietowski et al was 40.8% [10,21]. In Kato's series from Japan, 74% complained of pruritus [11]. Benchikhi et al reported a figure of 74.4% in HD patients from Moroc [22]. It is worth mentioning that a major drawback for studying and comparing results from different studies is the lack of a uniform way for assessment of this very subjective symptom. Recently, Yosipovitch et al has developed a comprehensive questionnaire for the assessment of pruritus and tested it in uremic patients and found it valid and reliable [23]. This questionnaire can provide data on many aspects of itching: the effect of daily life habits, physical activities and antipruritics on itch, the effect of itch on quality of life, temporal factors, location, severity, characteristics and exacerbating and relieving factors are all included. They found a significant correlation between the visual analogue scale and affective scores.

Uremic pruritus may be constant in 50% of cases with exacerbation or intermittent with spontaneous remissions. [24] In the majority of those affected, the itch is paroxysmal and may be localized (56%) or generalized (44%).[25] Seventy percent of our patients suffered from generalized pruritus as Moroccan patients.[22] Exacerbations of continuous itch are usually observed during HD or soon after, [1] which can be attributed to allergy to dialysate or dialysis membranes. [18] Exacerbation of pruritus during dialysis sessions was seen in 31.4% of HD patients in our study. In Yosipovitch series, too, itch appeared or aggravated in only 26% of patients during dialysis and the majority did not note an effect of dialysis on their itch. [23]

In Stahle-Backdahl et al's study, patients with pruritus tended to have been on dialysis treatment longer than those without pruritus.[2] Szepietowski et al showed a significant relationship between the total score of pruritus and duration of hemodialysis.[10] On the contrary, Altmeyer et al [26] described a significant improvement of itching in patients who had been on HD for long period of time: of the 23 patients with short term dialysis (2–3 years), 78% complained of pruritus, while it was seen in only 43% of 28 patients with long-term dialysis (>8 years). Murphy et al [27], however, could not confirm that pruritus decreases with progressive duration of dialysis treatment. In our study, we did not find any relationship between pruritus and duration of dialysis as seen in some previous studies. [7,19,26,27]

Xerosis is seen in the majority of patients on HD [1,4,7,8] and it may contribute to pruritus. [1,7,10,11,20] A relationship between the degree of xerosis and pruritus has been demonstrated in several studies, [1,10,20,28,29] although others failed to find an association between skin hydration and pruritus using a skin surface hygrometer. [11,30,31] Xerosis found in HD patients has been attributed to increased level of vitamin A in epidermis, atrophy of sebaceous and sweat glands and dysautonomia. [1,7,15] In our study the prevalence of xerosis in pruritic and non-pruritic patients was 61.4% and 66%, respectively, and we couldn't find any association between xerosis and pruritus.

Increased serum levels of magnesium, phosphorus and calcium have been proposed to be involved in uremic pruritus by some authors. [1,9,15,24] It has been suggested that an increased skin divalent ions concentration may lead to microprecipitation of calcium or magnesium phosphate, which may be the cause of pruritus. [1] On the other hand, the role of magnesium itself in the modulation of nerve conduction and release of histamine from mast cells was put forward. [1] While marked improvement of uremic pruritus with low dialysate calcium and magnesium has been reported [32,33], only a few studies showed a significant correlation between serum or skin divalent ion content and the presence of pruritus.[1,6] Recently, Momose et al, found increased calcium ion concentration in the deepest layers of the epidermis indicating a disrupted calcium ion gradient in the skin.[6] Like most studies [3,22,31], we couldn't find any association between serum calcium and phosphorus and uremic pruritus.

Hyperparathyroidism has been proposed by some as a cause of uremic pruritus.[8] Cases of disappearance of pruritus after parathyroidectomy support this theory.[1,4,8] This hormone can indirectly lead to metabolic alterations that subsequently cause pruritus.[1] On the other hand, a direct role for parathyroid hormone in causing uremic pruritus has been questioned because of the failure of intradermal injections of PTH analogs to cause pruritus, and because of negative immunohistochemical studies for PTH in skin biopsy specimens.[19]

Furthermore, no correlation between PTH levels and intensity of itching was found in most studies.[1,9,31] Although pruritus improved after parathyroidectomy in one of our cases, we couldn't find any relationship between serum PTH and pruritus in our study.

Iron deficiency anemia contributes to renal itch according to some authors, but we couldn't find any relationship between hematocrit level and uremic pruritus as shown in more recent studies[3,31].

Likewise, we couldn't find any association between HD pruritus and atopy. No previous research was recorded in the literature.

In our study there were also no significant difference between pruritic and non pruritic HD patients according to age, sex, underlying renal disease, serum alkaline phosphatase, BUN and creatinine similar to previous studies. [1,3,4,11,20-22,31]

Peripheral neuropathy affects up to 65% of patients starting dialysis. Some consider pruritus another manifestation of this neuropathy.[17] Contradictory data have been published about neurone-specific enolase fibers in the epidermis of HD patients. [2,34] Zakrzewska-Pniewska and Jedras found nervous dysfunction especially the somatic component related to pruritus in uremic patients. Pruritus was more frequent in patients with paresthesia in their study. Moreover the relationship between the severity of pruritus and the incidence of paresthesia was significant. [35]On the other hand, reports showing the efficacy of lidocaine [36,37], capsaicin [9,38,39], and gabapentin [40,41] in controlling uremic pruritus are in favor of a relationship between neuropathy and itching in HD patients. Yosipovitch et al reported paradoxical heat sensation an early sign of uremic sensory neuropathy. [42]

Neuropathy was significantly more frequent in patients with pruritus than without in our series, showing a figure of 63.8% versus 42.1% (pv < 0.05). These findings were not confounded by the presence of diabetics in the study group, as pruritus was not significantly different between diabetics and non-diabetics. Clinical neuropathy was significantly more frequent in HD patients with pruritus in Mesic's cases.[43] Winkelman et al, too, showed correlation between restless legs syndrome and pruritus. [44]

In conclusion, clinical neuropathy was the only significant finding in the pruritic group in our study. This finding justifies further research on nerve function and neurotransmitters in hemodialysis patients. Furthermore, therapeutic or preventive modalities targeting neuropathy may be effective in controlling the itch in hemodialysis patients.

## Authors' contributions

MA conceived the study, participated in its design and coordination, and drafted the manuscript.

GMR participated in the design and conduct of the study.

SN and KB performed the data collection and the statistical analysis.

DM drafted the manuscript.

All authors read and approved the final manuscript.

## Competing interests

The author(s) declare that they have no competing interests.

## Pre-publication history

The pre-publication history for this paper can be accessed here:


